# Characterization of the Differential Pathogenicity of Candida auris in a Galleria mellonella Infection Model

**DOI:** 10.1128/spectrum.00013-21

**Published:** 2021-06-09

**Authors:** Victor Garcia-Bustos, Amparo Ruiz-Saurí, Alba Ruiz-Gaitán, Ignacio Antonio Sigona-Giangreco, Marta Dafne Cabañero-Navalon, Oihana Sabalza-Baztán, Miguel Salavert-Lletí, María Ángeles Tormo, Javier Pemán

**Affiliations:** a Department of Internal Medicine and Infectious Diseases, University and Polytechnic La Fe Hospital, Valencia, Spain; b Severe Infection Research Group, Health Research Institute La Fe, Valencia, Spain; c Department of Pathology, Faculty of Medicine and Dentistry, University of Valencia, Valencia, Spain; d Department of Medical Microbiology, University and Polytechnic La Fe Hospital, Valencia, Spain; Broad Institute

**Keywords:** *Candida*, *Candida auris*, filamentation, pathogenicity, virulence

## Abstract

Candida auris is an emergent multidrug-resistant fungal pathogen considered a severe global threat due to its capacity to cause nosocomial outbreaks and deep-seated infections with high transmissibility and mortality. However, evidence on its pathogenicity and the complex host-pathogen interactions is still limited. This study used the *in vivo* invertebrate model in Galleria mellonella to assess its virulence, exploring the mortality kinetics, melanization response, and morphological changes after fungal infection compared to Candida albicans and Candida parapsilosis, with known high and low pathogenicity, respectively. All C. auris isolates presented less virulence than C. albicans strains but higher than that induced by C. parapsilosis isolates. Increased pathogenicity was observed in nonaggregative phenotypes of C. auris, while the melanization response of the larvae to fungal infection was homogeneous and independent of the causing species. C. auris was able to filament in the *in vivo* animal model G. mellonella, with aggregative and nonaggregative phenotypes presenting various pseudohyphal formation degrees as pathogenicity determinants in a strain-dependent manner. Histological invasiveness of C. auris mimicked that observed for C. albicans, with effective dissemination since the early stages of infection both in yeast and filamented forms, except for a remarkable respiratory tropism not previously observed in other yeasts. These characteristics widely differ between strains and advocate the hypothesis that the morphogenetic variability of C. auris is an indicator of its flexibility and adaptability, contributing to its emergence and rising worldwide prevalence.

**IMPORTANCE** Candida auris is an emergent fungus that has become a global threat due to its multidrug resistance, mortality, and transmissibility. These unique features make it different from other *Candida* species, but we still do not fully know the degree of virulence and, especially, the host-pathogen interactions. In this *in vivo* insect model, we found that it presents an intermediate degree of virulence compared to known high- and low-virulence *Candida* species but with significant variability between aggregative and nonaggregative strains. Although it was previously considered unable to filament, we documented *in vivo* filamentation as an important pathogenic determinant. We also found that it is able to disseminate early through the host, invading both the circulatory system and many different tissues with a remarkable respiratory tropism not previously described for other yeasts. Our study provides new insights into the pathogenicity of an emergent fungal pathogen and its interaction with the host and supports the hypothesis that its morphogenetic variability contributes to its rising global prevalence.

## INTRODUCTION

Since the first description of Candida auris, its incidence has dramatically risen, causing severe outbreaks of health care-related invasive infections with a high mortality rate (1–4). C. auris is noticeably different from most other *Candida* species, owing to its high resistance to antifungal agents and common disinfectants (5, 6), its unprecedented capacity to colonize patients long term and propagate in health care facilities (3, 7), its ability to survive for weeks on fomites and surfaces (1, 8), and the difficulties in its identification by phenotypical and biochemical methods (9). Furthermore, its emergence has been independent and simultaneous on several continents, including Africa, America, Asia, and Europe. Phylogenetic analyses have defined five major clades to date, wherein isolates clustered geographically and appeared nearly identical (2, 10, 11). Although the scientific community has devoted efforts to exploring the biological traits of this unique emergent fungus, there is still limited evidence of its pathogenicity and, especially, the complex host-pathogen interactions. Recently, Galleria mellonella has emerged as an alternative to conventional murine models for virulence studies, as its innate immune system at its cellular and humoral level is structurally and functionally similar to that of mammals (12, 13). Besides, the replicability of the model, favored by the possibility to use large numbers of larvae and its lesser ethical implications, has made G. mellonella a useful tool to study *Candida* species pathogenicity. Lately, several studies in this model have been performed to assess the virulence of C. auris infection (14–17).

Candida albicans is considered the most virulent species of the genus (13, 18), and, among other mechanisms, such as biofilm formation, filamentation is one of its main pathogenicity determinants. Although C. auris is thought to be able to form only rudimentary pseudohyphae, especially under stress circumstances (19, 20), some works with a relatively large number of strains have demonstrated a degree of virulence comparable to or even higher than that observed for C. albicans (14, 15). The presence of aggregative and nonaggregative phenotypes or even strains with different behaviors, as well as diversity in the expression of proteins related to biofilm formation, filamentation, and other virulence factors (14, 21–23), suggest heterogeneous pathogenicity. Aside from that, the *in vivo* host-pathogen interactions through the histopathological assessment of infected individuals are hardly known, both in animal models and humans.

This study aimed to evaluate the pathogenicity of C. auris strains isolated from clinical samples from a large Spanish hospital outbreak in the G. mellonella model compared to C. albicans and Candida parapsilosis, species with known high and low virulence, respectively. This was performed through the analysis of the mortality rate in survival assays and melanization assays and by histopathological assessment of the tissue invasion, host immune response, and fungal distribution *in vivo*.

## RESULTS

The characteristics of the 10 isolates of C. auris used in this study are presented in Table 1. All strains had been obtained from clinical samples, isolated both from blood cultures of medical and surgical critically ill patients with invasive infection and from cultures of epidemiological surveillance. In a previous work of our group (7), we analyzed 58 C. auris isolates (41 blood isolates and 17 epidemiological surveillance isolates) from 48 patients using the amplified fragment length polymorphism (AFLP) technique. The results suggested that our isolates are clonal, with an overall similarity of >96%. In addition, our isolates form a separate and distinct group, both from isolates from other countries and from phylogenetically close yeasts, and are only related to isolates from South Africa (clade III). These results were further confirmed by whole-genome sequencing (WGS) at the Mycology Reference Lab of the Carlos III Institute and by Chow et al. (10). The cj98 strain used in this work was sequenced in those works. Hence, our strains were assumed to belong to clade III. Clinical isolates from patients with invasive disease were tested for antifungal susceptibility. All were fluconazole resistant and echinocandin susceptible. C. auris strain Cj197 was considered resistant to amphotericin B by following the CDC tentative breakpoints (24).

**TABLE 1:** Characteristics of the C. auris strains used in this study^*a*^

Strain	Origin	Patient diagnosis	Department	Phenotype	MIC (mg/liter)									
					**AMB**	**5FC**	**FLU**	**ITR**	**VOR**	**CAS**	**ANI**	**MCF**	**POS**	**ISA**
Cj98	Blood	Polytrauma	SICU	Nonaggregative	0.25	0.12	>256	0.25	2	0.5	0.06	0.06	0.03	0.064
Cj104	Blood	Polytrauma	SICU	Aggregative	0.5	0.06	>256	0.125	2	0.03	0.125	0.06	0.06	NA
Cj173	Blood	Polytrauma	SICU	Aggregative	0.5	<0.06	>256	0.06	2	0.06	0.06	0.06	0.03	NA
Cj175	Blood	Status epilepticus	MICU	Nonaggregative	0.5	0.06	>256	0.125	2	0.03	0.125	0.06	0.06	NA
Cj197	Blood	Febrile neutropenia	Medicine	Nonaggregative	2	0.25	>256	0.25	4	0.5	0.5	0.25	0.06	0.064
Cj198	Blood	Pneumonia	MICU	Aggregative	0.25	0.06	>256	0.25	1	0.06	0.125	0.06	0.03	NA
312775	Blood	Endocarditis	MICU	Nonaggregative	0.5	0.06	>256	0.125	8	0.03	0.125	0.06	0.06	0.75
124819	Rectal	Respiratory failure + ECMO	MICU	Nonaggregative	0.5	<0.06	>256	0.06	0.03	0.03	0.06	0.03	0.015	0.25
182482	Inguinal	Liver Tx	MICU	Nonaggregative	0.5	<0.06	>256	0.06	0.03	0.03	0.03	0.03	0.015	0.5
253107	Pharyngeal	Multiple myeloma	MICU	Nonaggregative	0.5	<0.06	>256	0.06	0.03	0.03	0.125	0.03	0.015	0.25

a ECMO, extracorporeal membrane oxygenation; Tx, transplant; NA, not applicable.

### Galleria mellonella survival assays. (i) C. auris presents an intermediate degree of virulence between C. albicans and C. parapsilosis.

**FIG 1:**
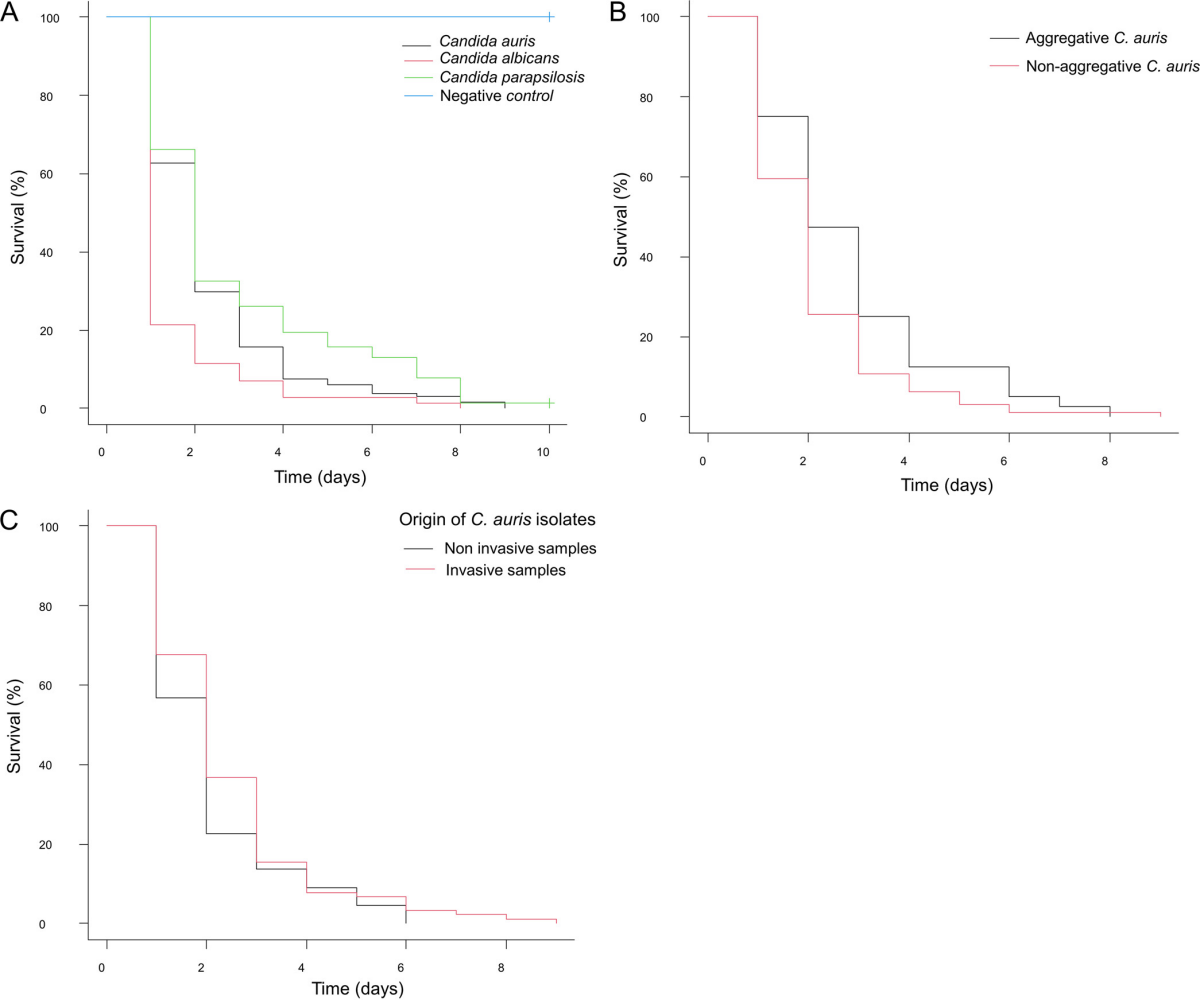
Kaplan-Meier survival curves of G. mellonella after infection with different *Candida* species. (A) Differences in global mortality kinetics between C. auris (2018-1-124819, Cj104, Cj98, 253107, 182482, 312755, Cj197, Cj198, Cj175, and Cj173), C. albicans (255083, Ca591, Ca581, Ca550, and Ca560), and C. parapsilosis (22019, 6308, Cp661, Cp664, Cp665, Cp669, Cp672, and Cp673) strains, including the negative control. (B) Differences in survival after infection with C. auris aggregative (Cj104, Cj173, and Cj198) and nonaggregative (2018-1-124819, Cj98, 253107, 182482, 312755, Cj175, and Cj197) phenotypes. (C) Differences in survival after infection with C. auris strains isolated from invasive samples (Cj98, Cj104, Cj173, Cj175, Cj197, Cj198, and 312775) and noninvasive samples (cultures of epidemiological surveillance) (124819, 182482, and 253107). All strains of different species, C. auris phenotypes, and clinical origins of C. auris were jointly assessed in the global analysis. Individual strain curves and data can be seen in the supplemental material.

Globally, significant differences were observed in the survival profile of G. mellonella larvae infected with C. albicans, C. auris, and C. parapsilosis species (*P* < 0.0001). The median survival was 48 h for C. auris and C. parapsilosis and 24 h for C. albicans, as seen in Fig. 1A. Larvae inoculated with C. albicans strains displayed significantly higher mortality rates than those infected with both C. auris (*P* = 0.00012) and C. parapsilosis (*P* < 0.0001). Moreover, C. auris strains showed higher virulence and produced lower survival rates than C. parapsilosis strains (*P* = 0.018). In all experiments, no larval deaths were documented in control groups inoculated with phosphate-buffered saline (PBS). The survival rates at half the observation time (120 h) were 6% (95% confidence interval [CI], 2.8 to 10.9) for C. auris, 2.9% (95% CI, 0.5 to 8.9) for C. albicans, and 15.6% (95% CI, 8.6 to 24.5) for C. parapsilosis. The Kaplan-Meier survival plots are represented in Fig. 1.

### (ii) Aggregative C. auris isolates are less pathogenic than nonaggregative isolates.

Aggregative and nonaggregative C. auris phenotypes were compared. While both presented a median survival of 48 h in infected larvae, nonaggregative C. auris strains were significantly more pathogenic than phenotypically aggregative strains (*P* = 0.0194) (Fig. 1B). The survival rates at half the observation time of aggregative and nonaggregative strains were 12.5% (95% CI, 4.6 to 24.6) and 3.2% (95% CI, 0.9 to 9.3), respectively. Both phenotypes were also differentially compared with C. albicans and C. parapsilosis. Nonaggregative strains exhibited significantly greater virulence than C. parapsilosis strains (*P* = 0.006) but still lower than that observed in larvae infected with C. albicans (*P* = 0.010), as also observed with aggregative strains (*P* = 0.00046).

### (iii) Clinical origin or drug resistance is not associated with increased virulence in C. auris.

No statistically significant differences were observed in the mortality induced by the amphotericin B-resistant C. auris strain Cj197, even after stratification by phenotype (*P* = 0.578). C. auris strains isolated from clinical samples of patients with invasive disease were not significantly more virulent than those obtained from epidemiological surveillance cultures, as represented in Fig. 1C (*P* = 0.172).

G. mellonella larvae did not show statistically significant differences in survival profiles in the penalized pairwise analysis, depending on each of the 10 individual isolates of C. auris they were infected with (*P* = 0.134), despite some interstrain variability. All C. auris strains induced significantly higher mortality rates than the negative control (*P* < 0.0001). The individual strains that seemed to be less virulent coincided with aggregative phenotypes (strains Cj104, Cj173, and Cj198). Similarly, no statistically significant differences were observed in the mortality rate after infection with the strains of C. albicans (*P* = 0.347). Wider differences could be observed in the interstrain pathogenicity of the other isolates of C. parapsilosis, although these were not statistically significant in our work either (*P* = 0.0678). All strains from both species were significantly virulent compared to the negative control (*P* < 0.0001). The survival Kaplan-Meier curves of all strains and nonstatistically significant comparisons can be found in the supplemental material.

### Melanization assays show the melanization response of G. mellonella to fungal infection seems to be independent of the infecting species.

**FIG 2:**
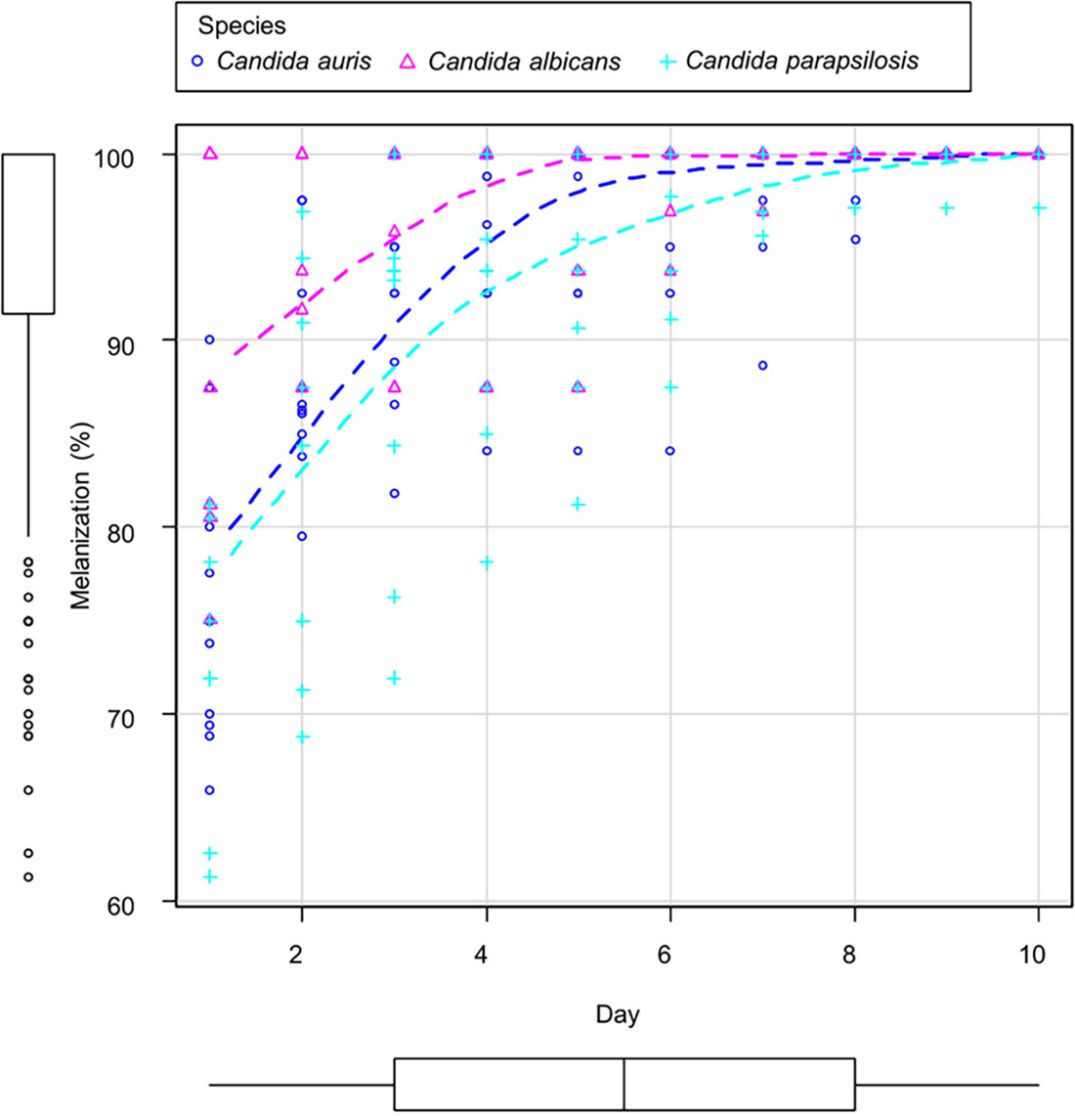
Scatterplot summarizing the melanization response of G. mellonella larvae after infection with different *Candida* species. The scatterplot represents the global mean percentage of larval melanization of each group of 10 larvae per strain for each day during the observation period, grouped by the different species. Smooth fitted lines are depicted for each species. All strains of the study were included in the analysis.

The degree of larval melanization was studied as a response to *Candida* species infection. In all three species, the melanization response in G. mellonella followed a logarithmic regression curve. The trend lines of the evolution of the mean larval melanization considering all strains of C. auris, C. albicans, and C. parapsilosis are represented in Fig. 2. No statistically significant differences were observed, as all three species induced similar melanization responses. However, as seen in Fig. 2, C. albicans strains seemed to cause the overall higher melanization rates, followed by C. auris and C. parapsilosis strains. Day four was chosen as the closer time to the inflection point of the curve. At this point, the mean degree of melanization in C. auris was 96.41% (standard deviations [SD], 5.29) in C. albicans was 95.54% (SD, 5.94) and in C. parapsilosis was 92.05% (SD, 8.77). To highlight, the reference C. albicans strains SC5314 and Ca550 induced severe and rapid melanization of up to 100% at 24 h. The nonaggregative C. auris strains Cj198 (87.5% at 24 h) and Cj175 (90% at 24 h) owned their group's higher melanization rates.

### Histopathology of infection in G. mellonella. (i) Fungal invasiveness, distribution, filamentation, and immune response after G. mellonella infection with C. auris.

**FIG 3:**
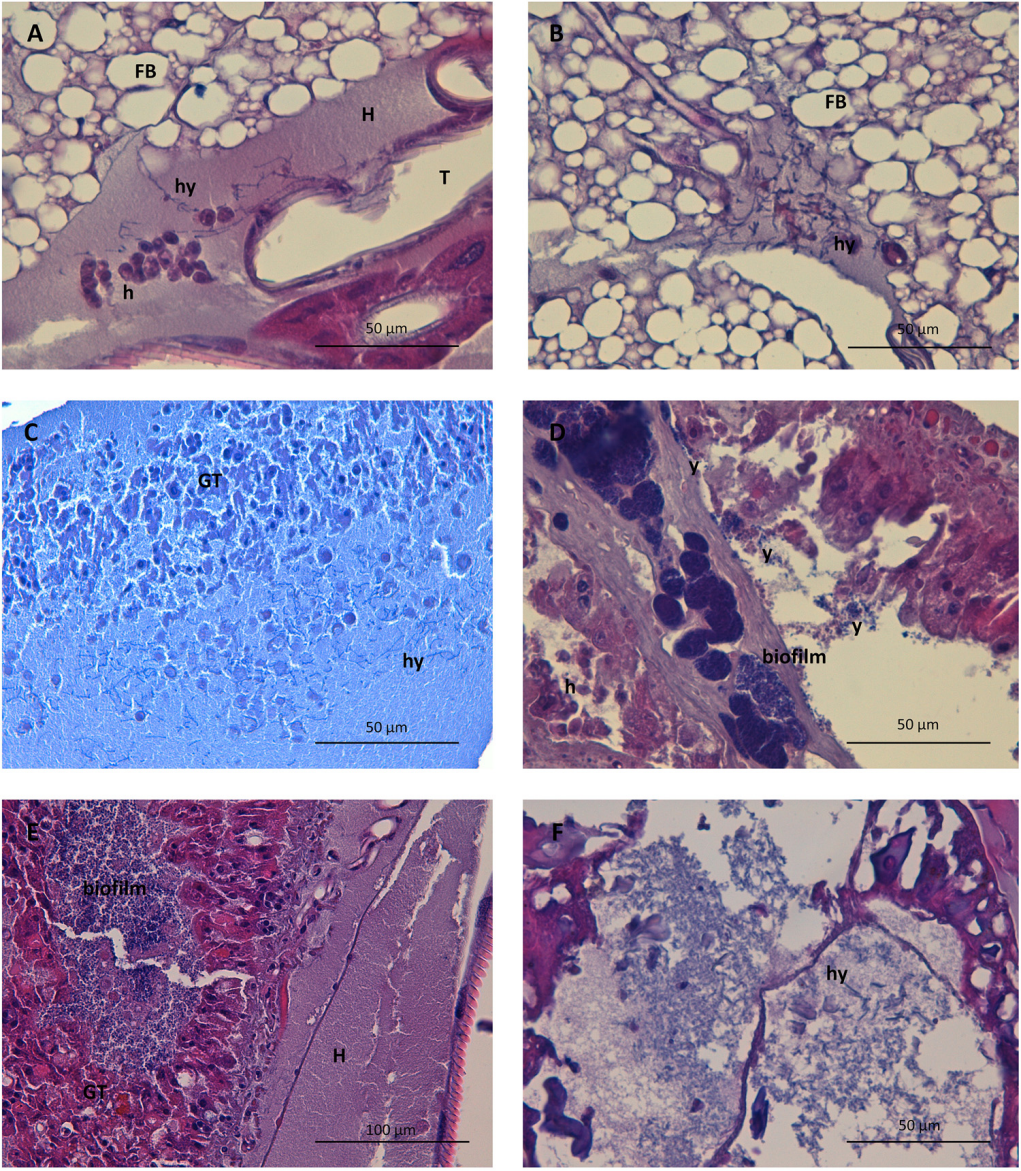
Representative photomicrographs of the fungal morphology and distribution in the G. mellonella model after infection with C. auris, C. albicans, and C. parapsilosis at different times. (A) Filamented C. auris disseminating through the hemolymph and invading the insect respiratory system twenty-four hours after infection with strain 253107. Periodic acid-Schiff (PAS) staining was used at ×630 magnification. (B) Invasion of the fat body by pseudohyphae of C. auris 24 h after infection with strain 253107. PAS staining is shown at ×630 magnification. (C) Pseudohyphae and yeasts of C. auris within the large granuloma-like formation of activated hemocytes of distinct lineages 48 h after infection with strain Cj175. Hematoxylin-eosin (H-E) staining is shown at ×630 magnification. (D) Large nodules of C. auris with yeast morphology and biofilm appearance within a matrix of detritus and inflammatory cells 24 h after infection with strain 253107. PAS staining is shown at ×630 magnification. (E) Granuloma-like formation with abundant necrosis surrounding significant accumulations of yeasts of C. albicans 48 h after infection with strain 255083. PAS staining is shown at ×400 magnification. (F) Yeast and filamented forms of C. parapsilosis 120 h after infection with strain 2209. PAS staining is shown at ×630 magnification. Abbreviations: FB, fat body; GT, granulation-like tissue; H, hemolymph; h, hemocytes; hy, pseudohyphae; T, tracheal system; y, yeasts.

**FIG 4:**
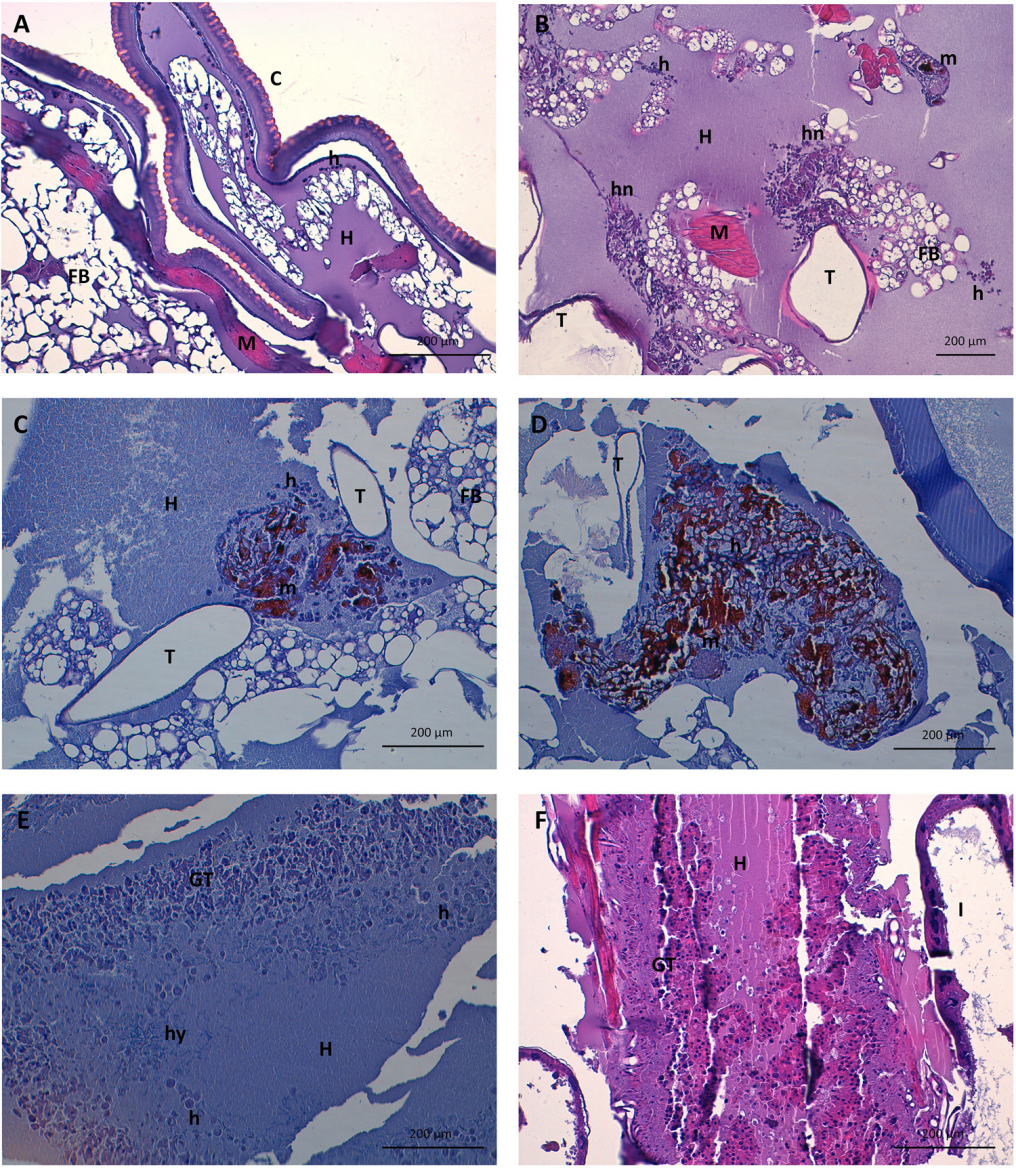
Histological response to fungal infection after inoculation of 10^6^ CFU/larvae of Candida auris. (A) Negative control. Note several isolated subcuticular hemocytes and conserved tissue architecture. Hematoxylin-eosin (H-E) staining is shown at ×200 magnification. (B) Inflammatory infiltrate conforming hemocyte nodules with peritracheal distribution of C. auris 48 h after infection with strain Cj104. H-E staining is shown at ×100 magnification. (C) Hemocyte nodule containing melanin deposits and possibly yeasts and pseudohyphae of C. auris with both peritracheal and hemolymphatic distribution 48 h after infection with strain Cj101. H-E staining is shown at ×100 magnification. (D) Large hemocyte nodule with abundant melanin deposits and fungal elements within after infection by C. auris at 48 h after infection with strain 175. H-E staining is shown at ×200 magnification. (E) Granulomatous tissue formed by activated hemocytes of different lineages and filamented structures of C. auris 48 h after infection with strain Cj175. H-E staining is shown at ×200 magnification. (F) Advanced stage of the infection by C. auris, with significant tissue destruction and necrosis and inflammatory response with granulation-like tissue 120 h after infection with strain Cj104. H-E staining is shown at ×200 magnification. Abbreviations: C, cuticle; FB, fat body; GT, granulation-like tissue; I, intestinal tract; T, tracheal system; H, hemolymph; h, hemocyte; hn, hemocyte nodule; hy, pseudohyphae; M, muscle; m, melanin; y, yeasts.

The fungal distribution, morphology, and burden was studied using periodic acid-Schiff (PAS), and the immune response was assessed with hematoxylin-eosin (H-E). In the C. auris infection model, significant dissemination was observed 24 h after inoculation, especially in nonaggregative strains. Mainly hemolymphatic dissemination was seen during these earlier stages, following a wider distribution throughout the tissue with a notable peritracheal involvement (Fig. 3A). *In vivo* infection induced pseudohypha formation in C. auris, mainly observed in hemolymph but also invading tissue and within granuloma-like formations (Fig. 3A to C). Various degrees of filamentation were observed in all strains, both in aggregative and nonaggregative phenotypes. Some strains, such as Cj175 or 253107, showed frequent and defined pseudohyphae, while in other isolates, such as Cj101, only rudimentary forms were observed. Large yeast aggregates with biofilm appearance were observed, especially in nonaggregative strains, within larger areas of disrupted and necrotic tissue with lower inflammatory response and with a time-dependent abundance (Fig. 3D). Increased numbers of activated hemocytes were seen in the inoculum site at 24 h. Hemocytes were later activated and recruited through various tissues; fewer subcuticular immature cells were observed, and activated histiocytic-like spindle cells were visualized in the fat body, digestive, and respiratory structures in later phases of the infection in surviving larvae (Fig. 4B to D). Significantly, hemocytes nodules, some containing fungal elements and melanin deposits, were formed dispersed in the fat body, frequently surrounding tracheae, but also intestinal walls and, in some cases, even muscular tissue (Fig. 4B to D). Even in the early stages, granuloma-like formations containing yeasts and pseudohyphae were found (Fig. 4E). Melanization bodies were subjectively larger and more frequent after infection by aggregative C. auris strains.

### (ii) Fungal invasiveness, distribution, filamentation and immune response after G. mellonella infection with C. albicans and C. parapsilosis.

C. albicans produced a faster and more aggressive progression of infection. Even in the earlier phases, the fungal elements reach solid organs, with significant tropism for the digestive tract. The infection also appears highly destructive and induced an intense inflammatory and even desmoplastic response, with granulation tissue containing highly abundant fungal elements with biofilm appearance (Fig. 3E). Forty-eight hours postinoculation, large amounts of fungal elements, both in yeast and filamented forms, distribute widely through the tissue, even distally to the inoculation point. In some strains, such as SC5314, marked filamentation is observed.

In the infection model with C. parapsilosis, both tissue invasion and inflammatory response are subjectively lower than that in larvae infected with C. auris and C. albicans, even in surviving larvae up to 120 h postinfection. Lesser solid-organ invasion is seen, and frequent fungal elements are seen in subcuticular regions and fat bodies. Granuloma-like formations were isolated and smaller than those observed in C. auris and C. albicans. Pseudohyphal forms were also found (Fig. 3F).

## DISCUSSION

The main findings can be summarized as follows. (i) C. auris isolates were less virulent than C. albicans strains but more virulent than C. parapsilosis isolates. (ii) Aggregative phenotypes of C. auris were less pathogenic than nonaggregative ones. (iii) C. auris, C. albicans, and C. parapsilosis induced similar rates of macroscopic melanization as a response to infection. (iv) C. auris can filament in the *in vivo* G. mellonella animal model. Both aggregative and nonaggregative phenotypes present pseudohyphal formation as pathogenicity mechanisms. (v) High tissue invasiveness is a general characteristic of C. auris. It mimics C. albicans but presents a general peritracheal distribution not previously described, inducing large granuloma-like formations and traveling both in yeast and filamented forms through liquid and solid compartments.

Since the discovery of C. auris (4), some works have been devoted to identifying its virulence and pathogenic mechanisms at both cellular and molecular levels due to its particular properties that are rarely observed in other yeasts (14–17), but the data are still limited and highly diverse. The understanding of its pathogenesis depends upon the highly complex host-pathogen interactions. The animal model G. mellonella is a useful tool for studying *Candida* species pathogenicity due to its high degree of structural and functional similarity with the innate immune system of mammals and has fewer ethical constraints (13, 25–29).

C. auris not only differs greatly from other species but also exhibits significant intraspecies heterogeneity, which evidences the need to include as many strains as possible as well as strains belonging to the different known clades (30–32). Therefore, to date, the degree of pathogenicity of C. auris compared to other *Candida* species is still under discussion. Moreover, the amount of *in vivo* studies and the number of strains used in the literature is still improvable. Furthermore, some studies present methodological limitations that constrain their external validity.

Borman and colleagues (14) used a relatively large number of United Kingdom isolates (12) and demonstrated strain-specific differences in C. auris pathogenicity. As in our study, aggregative phenotypes were less virulent than nonaggregative ones, as described previously (15). However, in contrast to our findings, the pathogenicity of nonaggregative isolates was similar to that observed in C. albicans. Nevertheless, despite the fact that Kaplan-Meier plots were created, the use of the Mann-Whitney test in larva survival comparison is debatable, and no *P* value penalization was applied in multiple comparisons, exponentially raising the probability of type 1 error. No histological assessment and no *in vivo* host-yeast interaction studies were performed. However, the global survival curves of C. auris in G. mellonella in their work are somehow similar to those obtained in our assays. Hence, despite there being statistically significant differences, its biological implications are unclear. Moreover, the use of this model serves as an initial approximation of the pathogenicity of C. auris in higher animals, but the validation of results will require further confirmatory mammalian testing. Strikingly, in the work by Sherry and coworkers (15) using 4 UK strains of C. auris, the C. auris strain SC5314 and C. glabrata WT2001, C. albicans and nonaggregating phenotypes of C. auris had similar kill kinetics with an inoculum of 10^6^ CFU, and the mortality induced by C. auris was even higher than that of C. albicans with a lower inoculum of 10^5^ CFU.

On the contrary, we reported lower mortality rates in larvae infected with C. auris than with C. albicans, in line with other studies, both in the different individual strains and phenotypes (16, 17). On the one hand, in experiments by Muñoz et al., the pathogenicity of 5 Colombian strains of C. auris was compared with the reference C. albicans strain SC5314 in the G. mellonella model and with the reference C. albicans strain ATCC 10231 in an immunosuppressed murine model as well as with strains of the *C. haemulonii* complex. Their findings in the G. mellonella model regarding their lower pathogenicity species control (*C. haemulonii* complex) are similar to ours concerning C. parapsilosis: in their study, C. auris strains showed significantly higher virulence than *C. haemulonii* complex species and lower pathogenicity than C. auris SC5314. In our work, significantly lower pathogenicity was observed in all strains of C. parapsilosis compared to C. albicans, as widely known (33), as well as C. auris. However, these results were not replicated in their murine model. Similar to previous evidence (15), a significantly higher capability of biofilm formation was seen in C. auris compared to the *C. haemulonii* species complex, and a higher fungal burden was also reported for C. auris. No distinction was made in this work between aggregative and nonaggregative phenotypes.

On the other hand, Romera et al. (16) used three Spanish strains of both C. auris and C. albicans, and an assessment of melanization, cocoon formation, and larva activity was also performed. However, no histological analyses were performed to corroborate results with findings regarding fungal morphology and behavior *in vivo* as well as the host immune system-yeast interactions. While their findings concur with our evidence regarding higher virulence observed in C. albicans, related to lower larval activity and cocoon formation but maintaining similar melanization levels, as in our case, no differences were observed in aggregative and nonaggregative phenotypes. Additionally, significant differences between clinical and reference strains were found in both species, probably owing to variations of the expression of virulence factors (16, 34, 35). In our work, despite all strains of C. auris being clinical, no differences were found in kill kinetics between strains isolated from invasive samples or cultures of epidemiological surveillance. This suggests that virtually all strains have the potential to provoke severe invasive disease independently of their isolation, as has been shown previously (27, 36).

Beyond the stated limitations of previous works and their mainly exploratory nature due to a lack of histological assessment and use of a small number of strains, the significant virulence heterogeneity between clades, phenotypes, and strains seems evident (32) and has been observed in other *Candida* species (33). C. auris has unique characteristics that make it different from other known yeasts and facilitate host infection and high environmental adaptation, and various virulence factors have been described to date. These variations in their pathogenicity determinants derived from the genomic plasticity of C. auris may explain this intraspecies heterogeneity.

Filamentation and biofilm formation, phenotypic switching, metabolic flexibility and pH adaptation, production of extracellular hydrolytic and cytolytic proteins, secretion of heat shock proteins, and adherence mechanisms are some of the major virulence determinants of *Candida* species (37, 38). Little is still known about C. auris virulence factors. Biofilm formation and hyphal growth have been considered the core factors enhancing the progression of pathogenicity in *Candida* spp. (37). Different authors have previously underlined the inability of C. auris to produce pseudohyphae both *in vitro* and in several models of infection, including G. mellonella, mouse, and human (1, 14, 17, 32, 39). This fact is distinctive, especially considering that filamentation has been linked to invasive ability into host tissues (40), and the lethality of invasive infections is up to 60% (2, 36, 41). However, it is evident that many other factors, such as diagnostic delay, multidrug resistance, or comorbidities, among others, are directly related to poorer outcomes in infected patients. Surprisingly, we have noted *in vivo* significant but divergent filamentation in both aggregative and nonaggregative phenotypes, ranging from rudimentary to well-defined pseudohyphae. These structures have been observed traveling free through the hemolymph, which raises the theory that dissemination to different organs through the circulatory system depends on normal yeast cells (20). Unlike C. albicans, which presents important gastrointestinal tropism in the G. mellonella model (25), pseudohyphae have been observed invading tissue with a remarkable peritracheal involvement in all strains of C. auris from both clinical invasive and epidemiological surveillance samples, which is not previously described in the literature for other yeasts, and within granuloma-like formations of spindle cells and activated hemocytes. However, this information is qualitative, and further quantitative or semiquantitative approaches must be performed to confirm this hypothesis in the insect model. Besides, insect models only represent the innate immune system and give no insight on the adaptive immune mechanisms of mammalians. Insect larvae lack many of the target organs of invasive fungal infections, and these data should be further replicated, at least in murine models. Recently, some studies have also reported filamentation in strains of C. auris under particular conditions or stress. Yue et al. described a heritable phenotypic switch to filamentation-competent/filamentous phenotype triggered by passage through the mammalian body and temperature changes. Bravo Ruiz and coworkers (19) were able to induce filamentation *in vitro* triggered by genotoxic stress. Remarkably, and in line with our work, Sherry et al. (15) described pseudohyphae in C. auris biofilms and Fan et al. (42) reported filamentation in strains of four major clades, both in the G. mellonella model. The fact that in some works no expression of hyphae-related proteins, such as candidalysin (ECE1) or hyphal cell wall protein (HWP1), has been detected (23), and that C. auris strains have differential biofilm-forming capability (15) or diversity in the expression of other virulence factors also seen in C. albicans (21, 22), suggests that the remarkable morphogenetic plasticity of C. auris is an indicator of its flexibility and adaptability and could contribute to its emergence and rising worldwide prevalence. Moreover, it questions the idea that each clade harbors nearly identical strains and emphasizes the importance of transcriptomic and proteomic analyses of different strains (2, 43, 44).

Our study presents several important limitations that need to be acknowledged. This is probably the most extensive study analyzing C. auris pathogenicity in terms of survival, melanization, and host-yeast histological interactions. However, the number of tested strains is relatively low and research is still needed, including more isolates of different morphologies, phenotypes, and clades due to the heterogeneous nature of the species. Our histological approach was qualitative, and larger and, more importantly, quantitative morphometric studies are needed to corroborate the results and analyze the immune response and fungal burden and distribution *in vivo*. Furthermore, other markers, such as larval activity and cocoon formation, have been described during the realization of this work. Genomic, proteomic, hemocyte count, and biofilm capacity analyses were not performed and are of interest in the study of fungal pathogenicity, especially in the unique context of C. auris.

In conclusion, G. mellonella is a useful model to evaluate the pathogenicity of emerging fungal pathogens such as C. auris. The virulence features of C. auris observed in this study widely differ between strains and clades. Further studies on pathogenicity mechanisms *in vitro* and *in vivo* are needed to keep unraveling the pathogenicity of this intriguing species.

## MATERIALS AND METHODS

### Fungal strains.

Ten strains of C. auris (2018-1-124819, Cj104, Cj98, 253107, 182482, 312755, Cj197, Cj198, Cj175, and Cj173), isolated both from blood cultures and epidemiological surveillance cultures of patients admitted to the University and Polytechnic Hospital La Fe (UPHLF), were selected in a randomized manner from the strain collection database of our institution.

The BacT/Alert Virtuo automated system (bioMérieux, Marcy l’Etoile, France) was used to process blood cultures. C. auris identification was performed in the Department of Microbiology of the UPHLF by sequencing the internal transcribed spacer (ITS) using the primers ITS3-ITS4 and ITS2-ITS5 with the GenomeLabTM GeXP system (Beckman Coulter, Fullerton, CA, USA). It was later confirmed in the Spanish Mycology Reference Laboratory using ITS1-ITS4 primers. Phenotypic classification of C. auris strains into aggregative and nonaggregative phenotypes was carried out by vigorous vortexing for 3 min with 1 ml of sterile saline containing a concentration of 10^8^ CFU/ml and immediate direct view of 10 μl of the solution at ×200 magnification using a TC20 automated cell counter (Bio-Rad Laboratories, France).

For the higher pathogenicity control model, six strains of C. albicans were chosen. Five (255083, Ca591, Ca581, Ca550, and Ca560) were obtained from blood cultures of patients from the UPHLF. One was isolated from cerebrospinal fluid (CSF) (Ca589), and one was the reference strain SC5314. For the lower pathogenicity control model, eight strains of C. parapsilosis (22019, 6308, Cp661, Cp664, Cp665, Cp669, Cp672, and Cp673), isolated from blood cultures of patients from the UPHLF, were used. Clinical isolates of C. albicans and C. parapsilosis species were identified in the Department of Microbiology of the UPHLF by using the matrix-assisted laser desorption ionization time of flight Vitek mass spectrometry system (bioMérieux, France). *In vitro* antifungal susceptibility was determined by EUCAST methodology. MIC was defined as the concentration achieving 50% growth inhibition after 24 h of incubation at 35°C (90% in the case of amphotericin B). MIC was determined by the colorimetric microdilution panel Sensititre YeastOne Y010 (TREK Diagnostic Systems, Oakwood Village, OH) according to the manufacturer’s instructions. The MIC to isavuconazole was determined by Etest isavuconazole Liofilchem MTS (Liofilchem, Roseto degli Abruzzi, Italy). CDC tentative breakpoints were applied for C. auris MIC interpretation (24).

All strains were kept at −80°C until use.

### Galleria mellonella.

Sixth-instar larvae of G. mellonella were acquired from the genome-sequenced breeding stock TruLarv (BioSystems Technology Ltd., UK). Nonmelanized active larvae weighing 250 to 350 mg were selected, and a first decontamination using 70% ethanol was carried out. Groups of 10 larvae were then placed in petri dishes and maintained at 15°C under dark conditions until inoculation.

### Survival assays in G. mellonella.

Survival assays in G. mellonella were performed by following previous protocols (14). Suspensions of *Candida* species isolates were grown on Sabouraud’s agar for 24 h at 37°C. Colonies were collected with sterile plastic loops, washed twice in sterile PBS, counted with a TC20 automated cell counter (Bio-Rad Laboratories, France), and adjusted to 10^5^ CFU/μl in sterile PBS. As Borman et al. (14) described, the obtaining of inocula of C. auris aggregate strains was performed by acquiring homogeneous suspensions by allowing initial suspensions to settle for 10 min, removing the supernatant containing individual yeasts, and then adjusting the concentration to 10^5^ CFU/μl. Before inoculation, larvae were further decontaminated with 70% ethanol. Individual larvae were infected by intrahemocelic injection of 10 μl of the solution (10^6^ CFU) in the left rear proleg using a 10-μl Hamilton syringe with a 26-gauge blunt needle. A minimum of 10 larvae per isolate were used in the cases of C. albicans and C. parapsilosis, and a minimum of 20 larvae per isolate were used in the cases of C. auris. Negative controls with sterile PBS were performed in the same way using groups of 10 larvae. Infected larvae were placed in petri dishes in groups of 10 and incubated at 37°C. Every petri dish was tagged with an individual code to blind the further scoring. Deaths and cocoons that occurred in the first 12 h after inoculation were discarded. Death of larvae was scored daily for 10 days.

### Melanization assays in G. mellonella.

The degree of melanization of the infected larvae in the survival assays was also monitored daily during the 10-day follow-up period. In each experiment, the percentage of body melanization was noted from 0 to 100% per larva and per day. The dead larvae were also recorded, scoring 100%. All processes were also blindly registered.

### Histopathology of G. mellonella.

Surviving larvae infected with each of the three species of *Candida* were euthanized after 24 h, 48 h, and 120 h. Euthanasia was performed in a 2-step method by following the AVMA guidelines for the euthanasia of animals (45). Larvae were first anesthetized through immersion in 5% ethanol and later euthanized by immersion in a solution of neutral-buffered 10% formalin to preserve tissue anatomy and start fixation. To perform an adequate fixation and maximum tissue preservation, larvae were maintained in this solution for 3 to 4 weeks to allow cuticular penetration and tissue diffusion. After fixation, they were sagittally sectioned and embedded in paraffin for processing. Conventional hematoxylin-eosin (H-E) staining was performed for the larvae's anatomical assessment and the immune response to infection. Periodic acid-Schiff staining (PAS) was performed to evaluate the fungal morphology and distribution, and Grocott-Gomori's methenamine silver stain was performed to confirm fungal morphology. Uninfected larvae were equally processed for comparative purposes. Histological samples were analyzed with a Leica DMD108 optic light microscope (Leica Microsystems, Wetzlar, Germany), and photomicrographs were taken at different magnifications.

### Statistical analysis.

The statistical analysis was performed with R statistical package version 4.0.3. (R Development Core Team, 2020). In the survival assays, Kaplan-Meier survival curves were created, and the log-rank test was used to compare larva survival in different groups. A *P* value of <0.05 was considered statistically significant. The Bonferroni adjustment method was applied for pairwise comparison. For the global comparison between species, all larvae from all strains of each species were included in the analysis, and the same methodology was performed depending on the grouping variables.

For the analysis of melanization assays, the mean degree of melanization for each observation day was calculated per strain and per experiment. A grouped scatterplot representing the global mean percentage of larval melanization of each group of 10 larvae per strain for each day during the observation period, grouped by the different species, was created, and smooth fitted lines were depicted. Day four after the infection was chosen according to the designed curves to analyze melanization rates. Normality was assessed using quantile-quantile plots, and the Kruskal-Wallis test by ranks was used.

The data set can be found in the Figshare repository with https://figshare.com/articles/dataset/Pathogenicity_auris_DB/14222456.

### Ethics statement.

The study protocol was approved by the Research Commission and the Scientific and Ethical Committees of the Health Research Institute La Fe with registry code 2017-0682. As the study was conducted in fungal strains and insect larvae and all clinical information of the origin of the samples was anonymous, no evaluation by the Institutional Animal Care Committee or informed consents were required.
