# Supplementary material for: Characterization of the Differential Pathogenicity of Candida auris in a Galleria mellonella Infection Model
Source: Microbiol Spectr. 2021 Jun 9;9(1):10.1128/spectrum.00013-21. doi: 10.1128/spectrum.00013-21 (PMC8552516; doi:10.1128/spectrum.00013-21)
Supplement: SUPPLEMENTAL FILE 1 — Fig. S1 to S5. Download SPECTRUM00013-21_Supp_1_seq7.pdf, PDF file, 1.2 MB [file spectrum00013-21_supp_1_seq7.pdf]

Supplemental material

S1. Detail of the Kaplan-Meier survival curves of Galleria mellonella infection with *C. auris, C. albicans* and *C. parapsilosis*

A. Differences in survival after infection with amphotericin B-resistant *C. auris* strain (cj197) and amphotericin-B susceptible *C. auris* (Cj 2018-1-124819, Cj104, Cj98, 253107, 182482, 312755, Cj198, Cj175, and Cj173). B, C and D: Differences in mortality kinetics of individual strains of *C. auris, C. albicans* and *C. parapsilosis,* respectively.

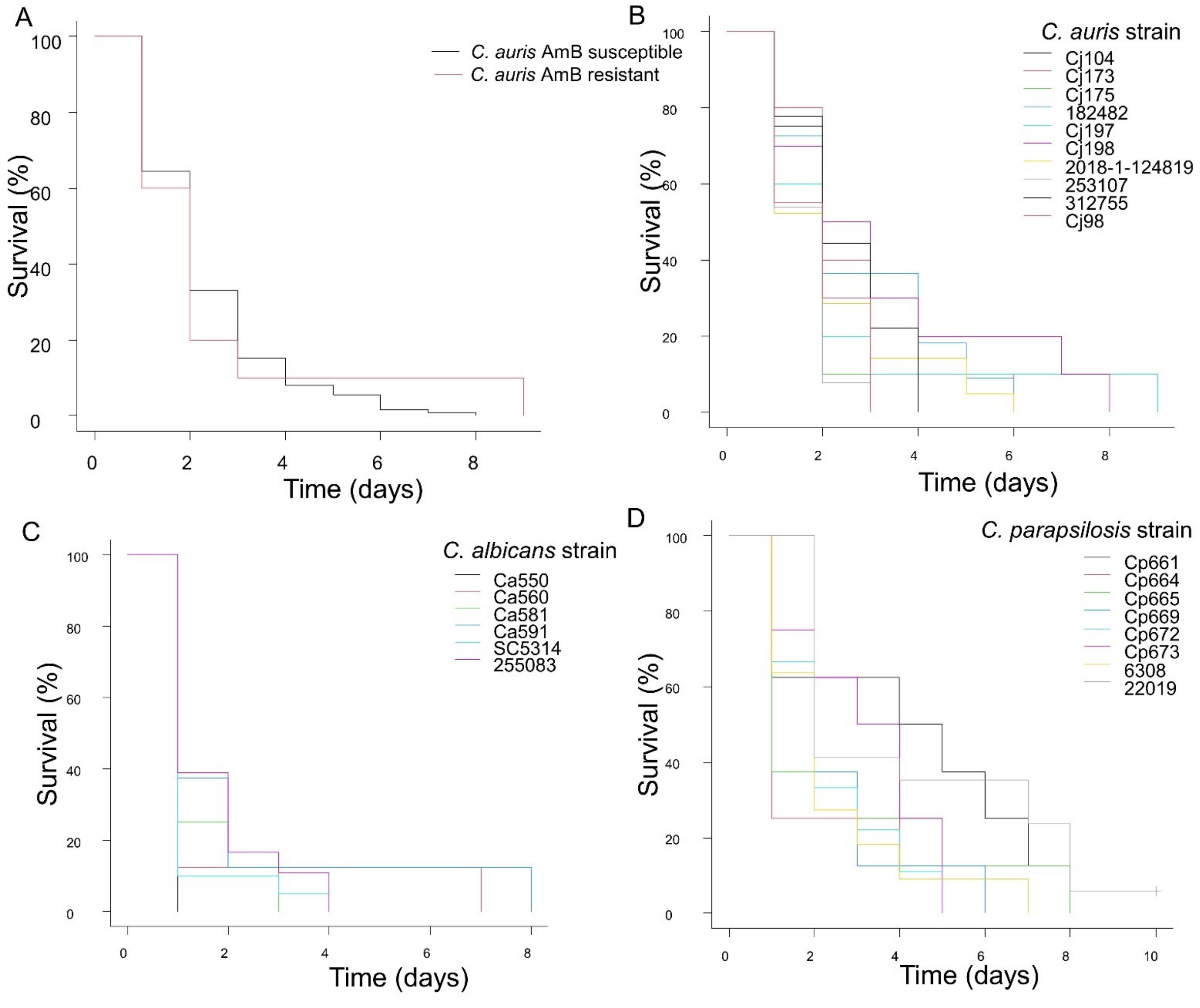

S2. Pseudohyphae and yeast form structures of *C. auris* with Grocott-Gomori's methenamine silver stain (GMS). A. Some pseudohyphal structures near respiratory and muscle tissue of *G. mellonella.* GMS. 630x magnification. B. Yeast structures tissue aggregates in *G. mellonella.* GMS. 630x magnification. M, muscle; T, tracheae.

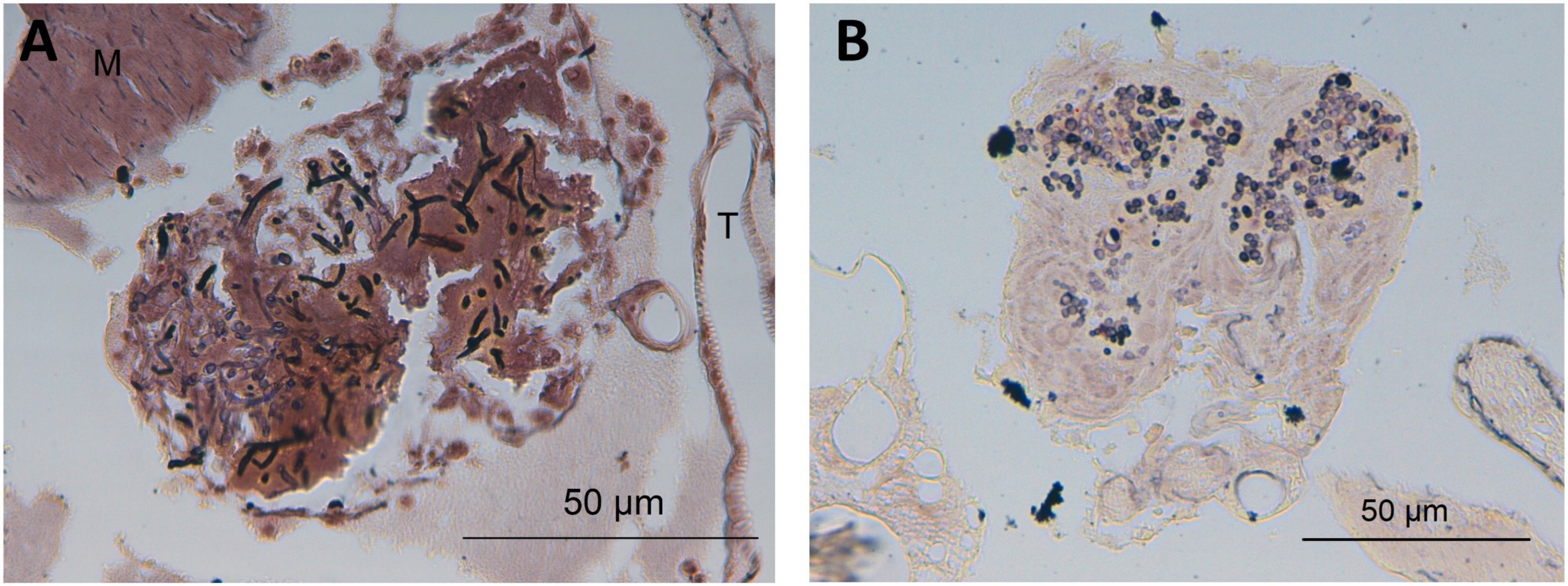
A
M
T
50 µm
B
50 µm

S3. Detail of Figure 3, A.

Filamented *C. auris* disseminating through the hemolymph and invading the insect respiratory system. 24 h after infection. Strain 253107. Periodic acid-Schiff (PAS) staining, 630x magnification. FB, fat body; H, hemolymph; h, hemocytes; hy, pseudohyphae; T, tracheal system.

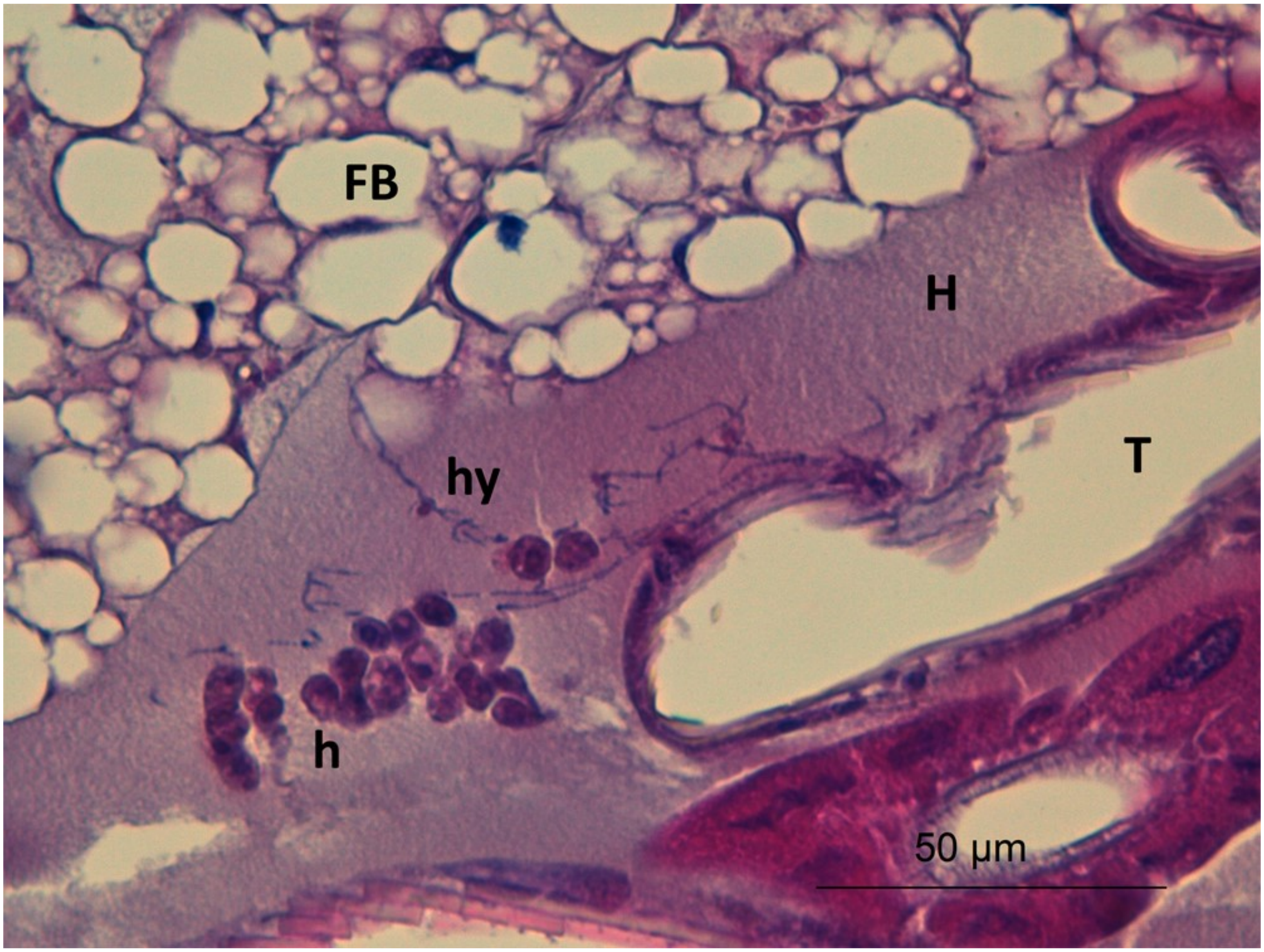

S4. Detail of Figure 3, B. Invasion of the fat body by pseudohyphae of *C. auris*. 24 h after infection. 24 h after infection. Strain 253107. PAS staining. 630x magnification. FB, fat body; hy, pseudohyphae.

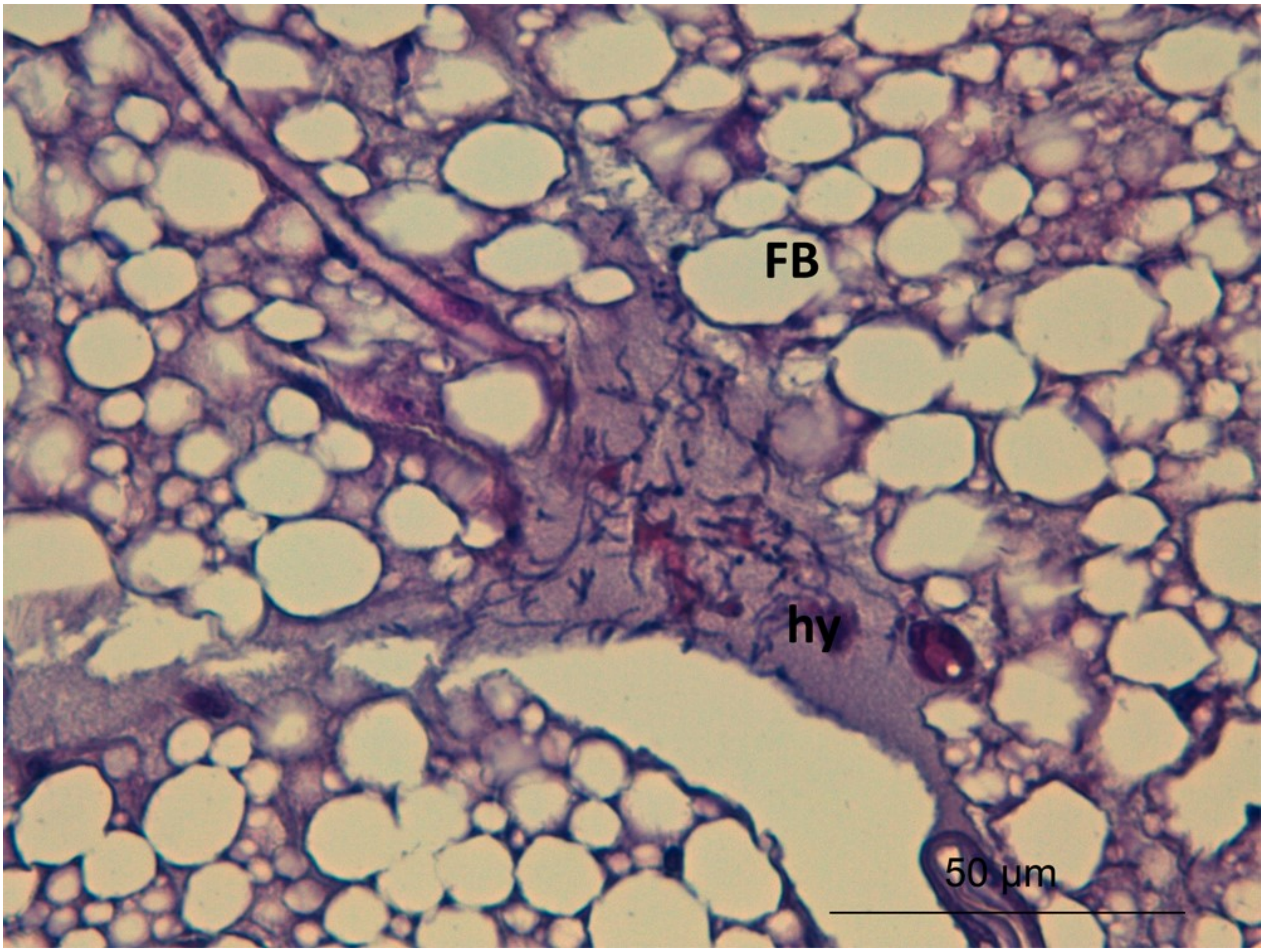

S5. Inter-strain differences of *G. mellonella* mean body melanization percentage per group of 10 larvae after infection with *C. albicans* (A), *C. auris* (B), and *C. parapsilosis* (C).

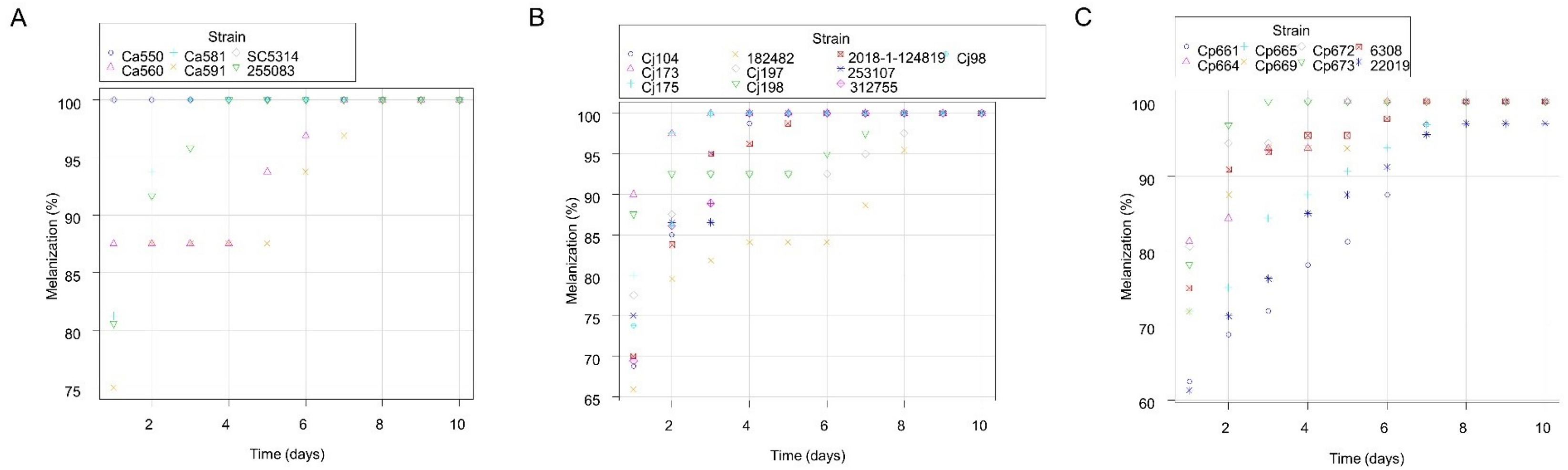
A
Strain
Ca550
Ca581
SC5314
Ca560
Ca591
255083
Melanization (%)
Time (days)
B
Strain
Cj104
182482
2018-1-124819
Cj98
Cj173
Cj197
253107
Cj175
Cj198
312755
Melanization (%)
Time (days)
C
Strain
Cp661
Cp665
Cp672
6308
Cp664
Cp669
Cp673
22019
Melanization (%)
Time (days)
